# How to reach agreement: the impact of different analytical approaches to Delphi process results in core outcomes set development

**DOI:** 10.1186/s13063-023-07285-1

**Published:** 2023-05-22

**Authors:** James Webbe, Benjamin Allin, Marian Knight, Neena Modi, Chris Gale

**Affiliations:** 1 Section of Neonatal Medicine, School of Public Health, Chelsea and Westminster Hospital Campus, Imperial College London, 369 Fulham Road, London, SW10 9NX UK; 2 National Perinatal Epidemiology Unit, University of Oxford, Oxford, UK; 3 Nuffield Department of Primary Care Health Sciences, University of Oxford, Oxford, UK

**Keywords:** Core outcomes sets, Delphi process, Consensus, Summary statistic

## Abstract

**Background:**

Core outcomes sets are increasingly used to define research outcomes that are most important for a condition. Different consensus methods are used in the development of core outcomes sets; the most common is the Delphi process. Delphi methodology is increasingly standardised for core outcomes set development, but uncertainties remain. We aimed to empirically test how the use of different summary statistics and consensus criteria impact Delphi process results.

**Methods:**

Results from two unrelated child health Delphi processes were analysed. Outcomes were ranked by mean, median, or rate of exceedance, and then pairwise comparisons were undertaken to analyse whether the rankings were similar. The correlation coefficient for each comparison was calculated, and Bland-Altman plots produced. Youden’s index was used to assess how well the outcomes ranked highest by each summary statistic matched the final core outcomes sets.

Consensus criteria identified in a review of published Delphi processes were applied to the results of the two child-health Delphi processes. The size of the consensus sets produced by different criteria was compared, and Youden’s index was used to assess how well the outcomes that met different criteria matched the final core outcomes sets.

**Results:**

Pairwise comparisons of different summary statistics produced similar correlation coefficients. Bland–Altman plots showed that comparisons involving ranked medians had wider variation in the ranking. No difference in Youden’s index for the summary statistics was found.

Different consensus criteria produced widely different sets of consensus outcomes (range: 5–44 included outcomes). They also showed differing abilities to identify core outcomes (Youden’s index range: 0.32–0.92). The choice of consensus criteria had a large impact on Delphi results.

**Discussion:**

The use of different summary statistics is unlikely to affect how outcomes are ranked during a Delphi process: mean, median, and rates of exceedance produce similar results. Different consensus criteria have a large impact on resultant consensus outcomes and potentially on subsequent core outcomes sets: our results confirm the importance of adhering to pre-specified consensus criteria.

**Supplementary Information:**

The online version contains supplementary material available at 10.1186/s13063-023-07285-1.

## Background

“Clinical trials are only as credible as their outcomes” [1]

Clinical trials guide clinical practice. They do this by demonstrating the beneficial or detrimental effects of an intervention to patients; these are the outcomes of the trial. When trial outcomes are not relevant to research users (patients, family members, and clinicians) statistically significant results may be clinically meaningless, and such trials will not necessarily translate into improvements in patient care [2, 3]. In many fields, the outcomes measured in clinical trials have been selected to meet the needs of researchers [4], rather than patients [5, 6]. One solution to these problems is the development of core outcomes sets; these also standardise outcome reporting, facilitating evidence synthesis and reducing outcome switching.

A core outcomes set is an agreed, standardised group of outcomes that it is recommended are reported by all trials within a research field [7]. Core outcomes sets are being developed across the spectrum of medical research [8]. A 2014 review identified 198 core outcomes sets [9], and this had increased to 366 by 2018 [10]. Core outcomes set development involves classifying them as more or less important so that outcomes that are crucial can be identified. A variety of different consensus methods have been used [9], but these can produce contrasting results. In paediatric asthma, a project relying on expert panel opinion identified different outcomes from a project that combined a Delphi process with patient and parent interviews [11, 12]. If core outcomes sets are to be widely adopted within different fields, researchers and clinicians need to have confidence in them; hence, they should be developed using robust methodology. There is no accepted definition of a ‘good’ core outcomes set [13], and if the wider research community identifies deficiencies in the included outcomes after the consensus process is completed, it will reduce uptake and limit utility [14].

The most common methodology used is a Delphi process that informs a subsequent consensus process, typically a consensus meeting [10]. The Delphi process involves participants answering serial surveys, with feedback on other participants’ scores provided between rounds [15]. While the methodology is increasingly standardised for core outcomes set development, there are still areas of uncertainty leading to variation in how Delphi processes are analysed and further research has been recommended [16, 17].

One area of uncertainty surrounds which summary statistics should be used during a Delphi process. A number of different summary statistics have been used including the mean [18] or median [19], while some projects have described the number of participants who scored outcomes above a certain threshold (referred to from this point onwards as ‘the rate of exceedance’) [20]. These summary statistics are used to give participants feedback on how outcomes were scored during previous rounds; this is a crucial step in the Delphi process that builds consensus between participants, but the optimal way to provide this feedback is unknown [17, 21]. If different summary statistics change the feedback participants are given, it could affect decision making, but there has not been an empirical analysis of how the summary statistic used influences the Delphi output.

Another area of uncertainty is how consensus should be defined in a Delphi process [17]. In other contexts such as research priority setting, it has been shown that using different criteria to define consensus can substantially alter Delphi results [22]. For core outcome set development, within a single Delphi study, it has been demonstrated that different consensus criteria influenced which outcomes are deemed ‘critical’ [23]. In this context, consensus criteria are intended to identify important outcomes to be discussed at the consensus meeting, but the relationship between ‘critical’ outcomes and those included in the eventual core outcomes sets has not been explored. Current guidelines specify that the consensus criteria used should be pre-specified [24] as there is a risk of bias if the criteria are changed after the Delphi results have been reviewed [16]. However, it has been noted that the choice of consensus criteria are rarely justified [13]; a review of Delphi studies found that the criteria used to define consensus vary widely [25]. The best-described criteria [14] are that, when scoring on a scale of 1 to 9, 70% of participants in each group should score an outcome as 7 to 9 with 15% or less scoring 1 to 3 [26]. The rationale is that this scoring pattern means the majority of participants view an outcome as being crucial with only a small minority dissenting [14], but these criteria have not been tested to assess how effectively they identify the outcomes that are included in final core outcomes sets.

This work aims to quantify the impact that different summary statistics and consensus criteria have on Delphi process results during core outcomes set development. We tested whether the use of different summary statistics affected outcome ranking and the degree to which the use of different consensus criteria influenced Delphi process results, and how these related to the final core outcomes sets identified in previous projects.

## Methods

We used data from two independent child-health Delphi studies that formed part of core outcomes sets development for gastroschisis [27] and neonatology [28]. We undertook two main analyses.

Firstly, to explore the effect of using different summary statistics, we calculated the mean, median, and the rate of exceedance (the number of participants who scored an outcome above a certain threshold) of a score of 7 for each outcome within each round of the two Delphi processes. We chose the threshold of 7 because in both studies, any score of 7–9 was interpreted as suggesting an outcome was ‘critical’ [27, 29]. Having calculated the summary statistics, we analysed how closely mean and median scores correlated. We then ranked the mean, median, and rates of exceedance for outcomes within each Delphi round and compared how well they correlated using Pearson’s correlation coefficient [30] for pairwise comparisons. As high correlation can reflect a wide variable range rather than true agreement, we also used these data to generate Bland–Altman plots [31]. Finally, we used each summary statistics to produce ‘consensus sets’ and compared whether these consensus sets matched the final core outcomes sets produced by the two processes. To ensure the consensus sets were the same size as the core outcomes sets, we limited the former to the top-ranked eight outcomes from the final round of the gastroschisis project and the top 12 from the neonatology project. Adapting a methodology used to assess how well a medical test separates diseased and non-diseased states, we calculated Youden’s index [32] to compare how well the different summary statistics predicted the final core outcomes set. Youden’s index specifies the probability that a test (in this case, the summary statistic) is informed in relation to the condition (in this case, the final core outcomes set) when compared to chance. A ‘perfect’ summary statistic that correctly ranked all of the final core outcomes set highest would have a Youden’s index of 1, while a summary statistic that ranked outcomes randomly with no relation to the final core outcomes set would have a Youden’s index of 0 [33]. Youden’s index has the advantage that it gives equal weight to false positives and false negatives and is independent of the relative sizes of the dichotomous groups. We compared Youden’s indexes using a *t*-test [32].

Secondly, we sought to evaluate the degree to which choice of consensus criteria influenced the outcomes selected as being ‘consensus’ by a Delphi process. We identified consensus criteria for comparison from a review [25] and applied these to the two Delphi processes described previously. We applied the criteria to the results of the final rounds from the two Delphi processes and considered how outcomes would be classified. We considered the outcomes to be ‘consensus’ if they met the criteria and ‘non-consensus’ if they did not. We then calculated the size of the resulting consensus sets and explored how closely the outcomes identified by each set of criteria matched the final core outcomes set for each project by calculating Youden’s index [32]. We compared Youden’s indexes using a *t*-test [32].

## Results

The two Delphi processes used were from gastroschisis [27] and neonatology [29]. Both core outcomes sets used a three-round Delphi process followed by a face-to-face consensus meeting, in line with the COMET handbook [13].

The core outcomes set for gastroschisis was developed using a Delphi process which contained 75 outcomes in round one and 87 outcomes in rounds two and three; eight outcomes were included in the final core outcomes set. It involved stakeholders from ten groups, which were combined into three panels for the Delphi survey: personal experience panel, neonatal panel, and non-neonatal panel (Supplementary Table S1). The consensus criteria used were as follows: “Over 70% of all participants score outcome 7–9 with less than 15% of all participants scoring an outcome 1–3”. The small number of participants in the researcher stakeholder group meant that it was impractical to apply the different consensus criteria to this group: their results were excluded from this analysis.

The core outcomes set for neonatology was developed using a Delphi process which contained 104 outcomes in round one and 114 outcomes in rounds two and three; twelve outcomes were included in the final core outcomes set. This had four stakeholder groups; former patients and parents, nurses and therapists, doctors, and researchers (Supplementary Table S2). The consensus criteria used were as follows: “Over 70% of participants in each group score outcome 7–9 with less than 15% scoring an outcome 1–3 in each group”.

We compared the mean and median scores for each outcome in each round and found strong correlation, *r* = 0.93 (Supplementary Figure S1). We then undertook pairwise comparisons of ranked means, ranked medians, and ranked rates of exceedance for the outcomes in the two Delphi processes (with ranking occurring within individual rounds of the processes). For all comparisons, strong correlation was seen with *r* > 0.9 (Table 1, Supplementary Figure S2, S3 and S4).

**Table 1:** Correlation coefficients for pairwise comparisons of ranked mean, median and rates of exceedance for Delphi outcomes

Comparison	COIN results	Gastroschisis results	Combined Delphi results
Ranked mean vs ranked median	0.92	0.95	0.94
Ranked mean vs ranked rates of exceedance	0.99	0.97	0.98
Ranked median vs ranked rates of exceedance	0.93	0.94	0.93

Pearson’s correlation coefficient calculated for pairwise comparisons

**Fig. 1:**
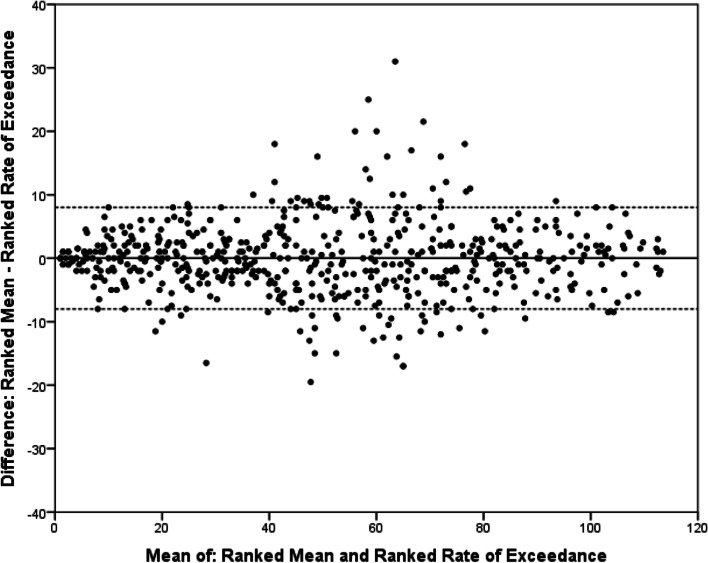
Bland–Altman plot comparing ranked mean scores and ranked rates of exceedance for outcomes across both Delphi projects. Mean and rate of exceedance calculated for each outcome and then ranked within individual rounds of the two Delphi projects. *X*-axis shows the mean of the two ranks for each outcome; *Y*-axis shows the difference between the two ranks for each outcome. Solid line represents the difference in mean ranking (*d* = 0). Dashed line represents upper and lower 95% limit of agreement (upper = 11.3, lower =  − 11.3)

**Fig. 2:**
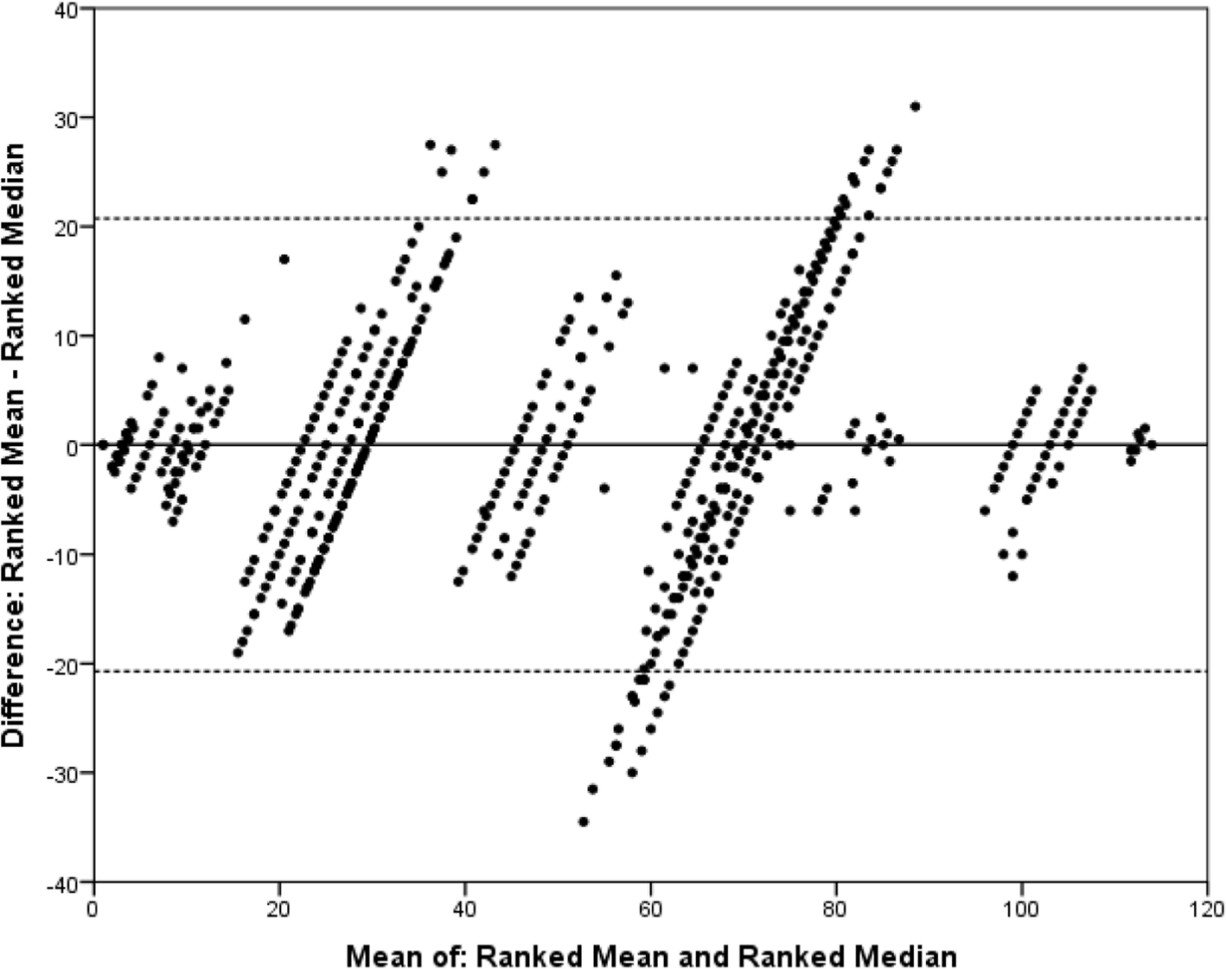
Bland–Altman plot comparing ranked mean scores and ranked median scores for outcomes across both Delphi projects. Mean and rate of exceedance calculated for each outcome and then ranked within individual rounds of the two Delphi projects. *X*-axis shows the mean of the two ranks for each outcome; Y-axis shows the difference between the two ranks for each outcome. Solid line represents the difference in mean ranking (*d* = 0). Dashed line represents upper and lower 95% limit of agreement (upper = 20.7, lower =  − 20.7)

**Fig. 3:**
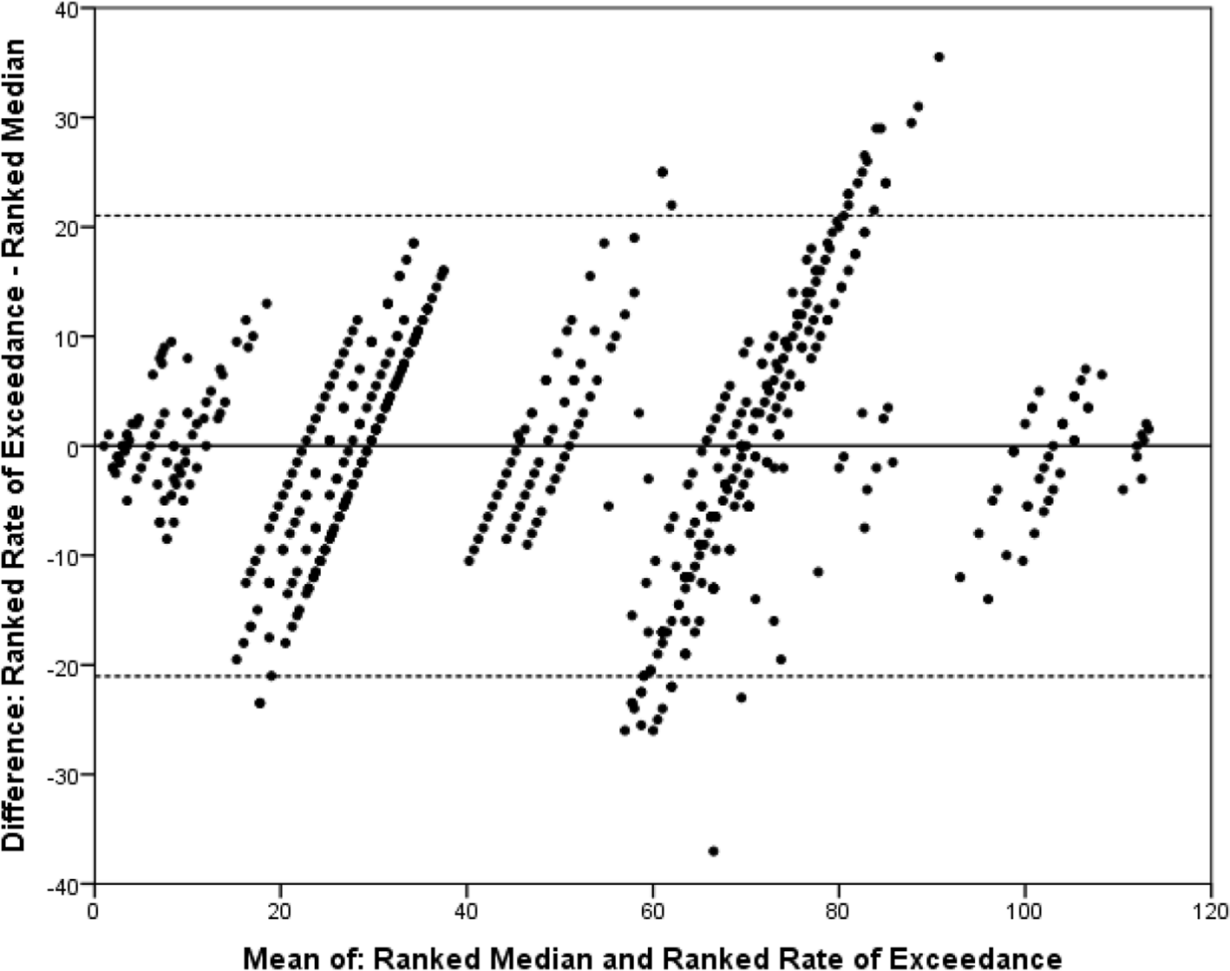
Comparison of ranked rates of exceedance and ranked median scores for outcomes across both Delphi projects. Mean and rate of exceedance calculated for each outcome and then ranked within individual rounds of the two Delphi projects. *X*-axis shows the mean of the two ranks for each outcome; *Y*-axis shows the difference between the two ranks for each outcome. Solid line represents the difference in mean ranking (*d* = 0). Dashed line represents upper and lower 95% limit of agreement (upper = 21.0, lower =  − 21.0)

We analysed the agreement between the summary statistics using the technique described by Bland and Altman [31]. These comparisons showed that the variation in rank differed less between ranked means and ranked rates of exceedance than between the ranked medians and the two other summary statistics (Figs. 1, 2, 3). As the plots relate to ranked summary statistics, with identical numbers of total ranks, for all comparisons, the mean difference is zero. These plots show that across all comparisons the agreement is best for the highest-ranked outcomes, with most disagreement seen for middle-ranked outcomes.

We examined how the use of different summary statistics influenced the Delphi output in relation to the final Delphi results.

We looked at the outcomes ranked highest in the final round of each Delphi process using the different summary statistics (Supplementary Table S3, Table 2) and compared how well the top-ranked outcomes predicted the final core outcomes set. There was no significant difference between the Youden’s index calculated for each summary statistic within each core outcomes set, although the summary statistics were all less predictive for the gastroschisis set.

**Table 2:** Ability of different summary statistics to correctly discriminate between outcomes included and excluded from the final core outcomes set

Summary statistic top-ranked outcomes	Youden’s index	
	Gastroschisis core outcomes set (*n* = 87)	Neonatal core outcomes set (*n* = 114)
Ranked mean score^b^	0.31 *(− 0.03, 0.65)*	0.81 *(0.60, 1.03)*
Ranked median score^a^	0.21 *(− 0.09, 0.51)*	0.79 *(0.58, 1.00)*
Ranked rate of exceedance	0.31 *(− 0.03, 0.65)*	0.91 *(0.75, 1.06)*

95% confidence intervals given in brackets

^a^Participants in the gastroschisis core outcomes set development project were presented with median scores during the Delphi process

^b^Participants in the neonatal core outcomes set development project were presented with mean scores during the Delphi process

To compare different consensus criteria, we identified nine sets of criteria:

- Allin et al.: Over 70% of all participants score outcome 7–9 with less than 15% all participants scoring an outcome 1–3 [27]
- Beattie et al.: Over 80% of all stakeholders score outcome 6–9 [34]
- Bennett et al.: Over 75% of all stakeholders score outcome 7–9 [35]
- De Lima et al.: Median score for all groups being between 7 and 9 [36]
- Playfor et al.: Over 90% of all participants scored an outcome over 7 [37]
- Qureshi et al.: Mean score for all groups being greater than 7 [38]
- Schmitt et al.: Over 60% of participants in 3 out of 4 groups score outcome 7–9 (with at least one of the groups being consumers) [39]
- Williamson et al.: Over 70% of participants in each group score outcome 7–9 with less than 15% scoring an outcome 1–3 in each group [7]
- Wylde et al.: Over 70% of participants in each group score outcome 7–9 or 90% of participants in any group score outcome 7–9 with less than 15% scoring an outcome 1–3 in each group [26]

We applied these consensus criteria to the results of the Delphi processes described previously. The size of the consensus sets produced varied from 5 to 44 included outcomes; the largest consensus sets contained up to 45% of the outcomes included in the Delphi process (Table 3).

**Table 3:** Size of core outcomes sets produced using different consensus criteria

Consensus criteria	Size of consensus set produced when definition applied to Delphi results	
	Gastroschisis core outcomes set (*n* = 87)	Neonatal core outcomes set (*n* = 114)
Allin et al.^a^	27 *(31%)*	24 *(21%)*
Beattie et al	39 *(45%)*	44 *(39%)*
Bennett et al	25 *(29%)*	21 *(18%)*
De Lima et al	39 *(45%)*	44 *(39%)*
Playfor et al	7 *(8%)*	5 *(4%)*
Qureshi et al	26 *(30%)*	24 *(21%)*
Schmitt et al	33 *(38%)*	29 *(25%)*
Williamson et al.^b^	18 *(21%)*	15 *(13%)*
Wylde et al	27 *(31%)*	20 *(18%)*

Number in italics is the percentage of outcomes in the final Delphi round that met the consensus definition

^a^Criteria used in the gastroschisis core outcomes set development project

^b^Criteria used in the neonatal core outcomes set development project

We also explored how well the different definitions identified outcomes found in the final core outcomes set. We calculated Youden’s index for each definition for each study (Table 4). These ranged from 0.92 to 0.32. All of the definitions performed worse when applied to the results of the gastroschisis core outcomes set. There was no definition that discriminated perfectly between core and non-core outcomes, and the best performing definition differed between the two studies.

**Table 4:** Ability of criteria to correctly discriminate between outcomes included and excluded from the final core outcomes set

Consensus criteria	Youden’s index	
	Gastroschisis core outcomes set (*n* = 87)	Neonatal core outcomes set (*n* = 114)
Allin et al.^a^	0.62 *(0.37, 0.87)*	0.88 *(0.82, 0.94)*
Beattie et al	0.46 *(0.21, 0.72)*	0.69 *(0.60, 0.78)*
Bennett et al	0.51 *(0.19, 0.82)*	0.91 *(0.86, 0.97)*
De Lima et al	0.60 *(0.49, 0.71)*	0.69 *(0.60, 0.78)*
Playfor et al	0.32 *(− 0.02, 0.66)*	0.42 *(0.14, 0.70)*
Qureshi et al	0.49 *(0.18, 0.81)*	0.88 *(0.82, 0.94)*
Schmitt et al	0.54 *(0.29, 0.79)*	0.83 *(0.76, 0.91)*
Williamson et al.^†^	0.46 *(0.11, 0.80)*	0.88 *(0.72, 1.03)*
Wylde et al	0.34 *(− 0.01, 0.69)*	0.92 *(0.87, 0.98)*

95% confidence intervals given in brackets

^a^Criteria used in the gastroschisis core outcomes set development project

^b^Criteria used in the neonatal core outcomes set development project

## Discussion

We show that the use of means, medians, or rates of exceedance is unlikely to affect how outcomes are ranked during a consensus process. However, different consensus criteria have a large impact on the outcomes produced by a Delphi process. The number of outcomes that meet different criteria varies substantially as does the ability of the latter to predict the outcomes that will form the final core outcomes set. As the criteria used will influence the outcomes discussed in the consensus meeting, and thus potentially influence the final set, our work reiterates the importance of adhering to pre-specified consensus criteria.

The importance of using pre-defined consensus criteria is recognised in current guidance [13, 24], but it has been found that consensus criteria are changed during some consensus processes [40]. Our findings replicate the previous finding that different criteria will identify different numbers of outcomes as critical [23], and for the first time, we have identified differences in how well they predict the final core outcomes set. In contrast, our work suggests that the impact of different summary statistics has been overstated. The choice of summary statistic has only a minimal impact on which outcomes are ranked as more or less important. Theoretical justifications have been given for using particular summary statistics: it has been suggested that the median is most appropriate because Likert scale data should be considered ordinal [41] and Delphi results are often skewed [21]. However, other researchers have both used the mean and recommended its use as standard analytic practice [42–44]. Despite theoretical differences, we demonstrate the impact of the use of different summary statistics is minimal in the context of core outcomes set development where the aim is to identify exceptional outcomes that are viewed as most important to all groups. Agreement between the summary statistics was best for the highest-ranked outcomes, and all summary statistics were similarly predictive of the final sets.

The strengths of our work include the application of statistical methods to data from two Delphi projects in unrelated research fields. Previous guidance in this area has primarily been based on theoretical considerations [42] or a priori statements [7], but we explored how different analytical approaches affect real-world results. Another strength is the range of pragmatic consensus definitions that we identified and compared: these have all been used in previous consensus projects. The main limitation is that we have had to use the final core outcomes sets as a ‘gold standard’. These sets will have been influenced by the particular summary statistics and consensus criteria used during their development, and the Delphi process results were further interpreted during the face-to-face consensus meetings before the core outcomes sets were agreed. While the methodology used to identify core outcomes sets is still being developed, and the conduct of consensus meetings is an area of particular uncertainty [13, 45], there is no other established way of identifying which are genuinely the most important outcomes in these fields. Using the core outcomes sets as the ‘gold standard’ could be expected to compromise the internal validity of this analysis as the statistics and criteria used to develop these sets might appear better than other approaches (leading to confirmation bias). However, our results suggest that using alternative methodologies during the Delphi processes would have produced results more predictive of the final core outcomes sets. Repeating this analysis with a larger number of methodologically different Delphi processes would reduce this internal confirmation bias. Having data from only two Delphi processes also means that our ability to identify the consensus criteria that perform best is limited. Repeating the same analysis with data from more Delphi processes might help ensure that any recommended consensus criteria are sufficiently generalisable to apply to all future work.

Current guidance recommends that as part of core outcomes set development a face-to-face consensus meeting is held to interpret the results of the Delphi process [13]. While there is increasing standardisation of Delphi methodology, the optimal format of these face-to-face consensus meetings is unclear, and there are differences of opinion over fundamental issues such as whether patients should be included [46] or should have a separate meeting [20]. The anonymity of participants and iterative approach of the Delphi methodology prevents distortion of the consensus process by dominant individuals with particular agendas [47]; interpreting Delphi results at a poorly conducted consensus meeting may undermine the benefits of the process. Our results show that the consensus criteria used are likely to have a large impact on the final consensus meeting: if too stringent criteria are used few outcomes may be discussed and essential outcomes might be missed, conversely loose criteria may mean that there is insufficient time for the detailed discussion needed. How researchers should conduct these meetings is beyond the scope of this work but identifying and implementing optimal consensus criteria would ensure that the Delphi results contribute in a more standardised way. The increasing numbers of core outcomes sets in development [8] require robust and consistent methodology to ensure that their results are reliable and deliver the intended benefits.

## Conclusions

The use of different summary statistics is unlikely to affect how outcomes are ranked during a Delphi process: mean, median, and rates of exceedance produce similar results. Different consensus criteria have a large impact on resultant consensus sets; at present, it is unclear whether an optimal definition exists. Consensus criteria should be pre-defined to prevent distortion of the Delphi process.

## Supplementary Information

**Additional file 1: Table S1.** Stakeholder participation across Delphi rounds in gastroschisis core outcomes set development project. **Table S2.** Stakeholder participation across Delphi rounds in neonatal core outcomes set development project. **Figure S1.** Comparison of mean and median scores for outcomes across both Delphi projects. **Figure S2.** Comparison of ranked mean and ranked median scores for outcomes across both Delphi projects. **Figure S3.** Comparison of ranked mean and ranked rates of exceedance for outcomes across both Delphi projects. **Figure S4.** Comparison of ranked rates of exceedance and ranked median scores for outcomes across both Delphi projects. **Table S3.** Outcomes ranked highest by different summary statistics in final round of gastroschisis core outcomes set development. **Table S4.** Outcomes ranked highest by different summary statistics in final round of neonatal core outcomes set development.

## Data Availability

All data generated during this study are included in this published article and its supplementary information files.
