# Supplementary material for: How to reach agreement: the impact of different analytical approaches to Delphi process results in core outcomes set development
Source: Trials. 2023 May 22;24:345. doi: 10.1186/s13063-023-07285-1 (PMC10201748; doi:10.1186/s13063-023-07285-1)
Supplement: Supplementary file 1 — Additional file 1: Table S1. Stakeholder participation across Delphi rounds in gastroschisis core outcomes set development project. Table S2. Stakeholder participation across Delphi rounds in neonatal core outcomes set development project. Figure S1. Comparison of mean and median scores for outcomes across both Delphi projects. Figure S2. Comparison of ranked mean and ranked median scores for outcomes across both Delphi projects. Figure S3. Comparison of ranked mean and ranked rates of exceedance for outcomes across both Delphi projects. Figure S4. Comparison of ranked rates of exceedance and ranked median scores for outcomes across both Delphi projects. Table S3. Outcomes ranked highest by different summary statistics in final round of gastroschisis core outcomes set development. Table S4. Outcomes ranked highest by different summary statistics in final round of neonatal core outcomes set development. [file 13063_2023_7285_MOESM1_ESM.docx]

**Online only supplemental material**

Table S1: Stakeholder participation across Delphi rounds in gastroschisis core outcomes set development project

Table S2: Stakeholder participation across Delphi rounds in neonatal core outcomes set development project

Figure S1: Comparison of mean and median scores for outcomes across both Delphi projects

Figure S2: Comparison of ranked mean and ranked median scores for outcomes across both Delphi projects

Figure S3: Comparison of ranked mean and ranked rates of exceedance for outcomes across both Delphi projects

Figure S4: Comparison of ranked rates of exceedance and ranked median scores for outcomes across both Delphi projects

Table S3: Outcomes ranked highest by different summary statistics in final round of gastroschisis core outcomes set development

Table S4: Outcomes ranked highest by different summary statistics in final round of neonatal core outcomes set development

**Table S1** Stakeholder participation across Delphi rounds in gastroschisis core outcomes set development project

| **Stakeholder Group** | **Round 1** | | **Round 2** | | **Round 3** | |
| --- | --- | --- | --- | --- | --- | --- |
|  | Started | Completed | Started | Completed | Started | Completed |
| Neonatal panel | 58 | 52 | 47 | 47 | 43 | 43 |
| Non-neonatal panel | 8 | 8 | 7 | 7 | 6 | 6 |
| Personal experience panel | 98 | 42 | 31 | 31 | 22 | 22 |
| Total | 164 | 102 | 85 | 85 | 71 | 71 |

The three Delphi survey panels included stakeholders from the following groups:

Neonatal panel: Paediatric surgeons. Neonatologists. Fetal medicine specialists. Specialist nurses. Specialist paediatric surgical nurses.

Non-neonatal panel: Paediatricians. Researchers. Dietitians.

Personal experience panel: People born with gastroschisis. Parents of children born with gastroschisis.

**Table S2** Stakeholder participation across Delphi rounds in gastroschisis core outcomes set development project

| **Stakeholder Group** | **Round 1** | | **Round 2** | | **Round 3** | |
| --- | --- | --- | --- | --- | --- | --- |
|  | Started | Completed | Started | Completed | Started | Completed |
| Parents and Patients | 244 | 111 | 84 | 61 | 61 | 53 |
| Neonatal nurses and allied professionals | 53 | 44 | 39 | 38 | 34 | 33 |
| Doctors | 83 | 74 | 71 | 62 | 67 | 59 |
| Neonatal researchers | 34 | 31 | 29 | 26 | 29 | 28 |
| Total | 414 | 260 | 223 | 187 | 191 | 173 |


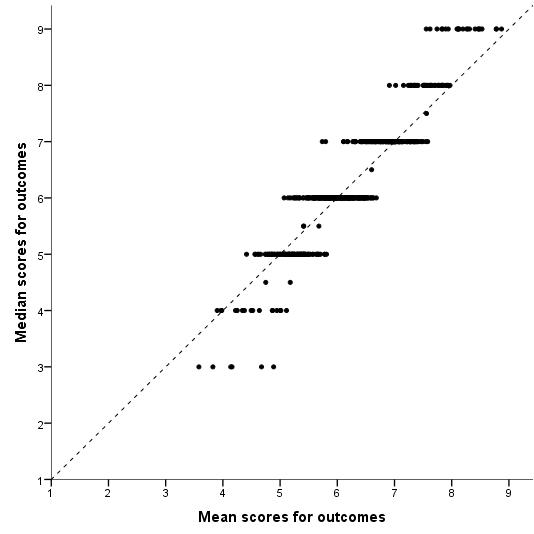
**Figure S1** Comparison of mean and median scores for outcomes across both Delphi projects

Mean and median calculated for each outcome within individual rounds of the two Delphi projects

The dashed line is the line of equality

**
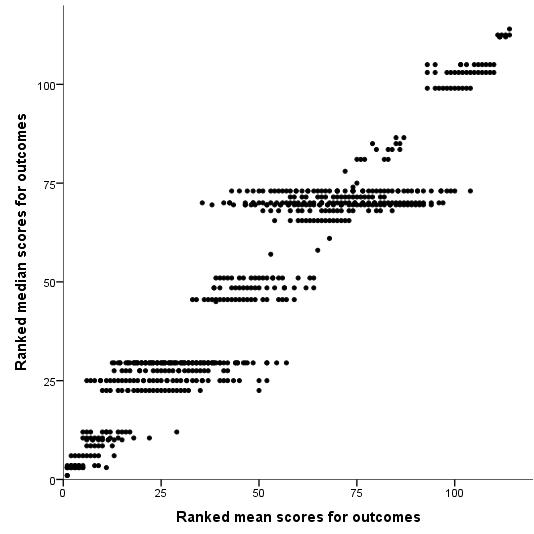
Figure S2** Comparison of ranked mean and ranked median scores for outcomes across both Delphi projects

Mean and median calculated for each outcome and then ranked within individual rounds of the two Delphi projects

**
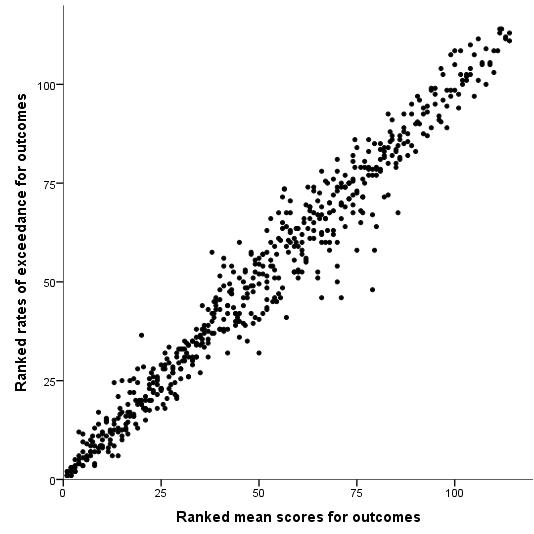
Figure S3** Comparison of ranked mean and ranked rates of exceedance for outcomes across both Delphi projects

Mean and rate of exceedance calculated for each outcome and then ranked within individual rounds of the two Delphi projects

**
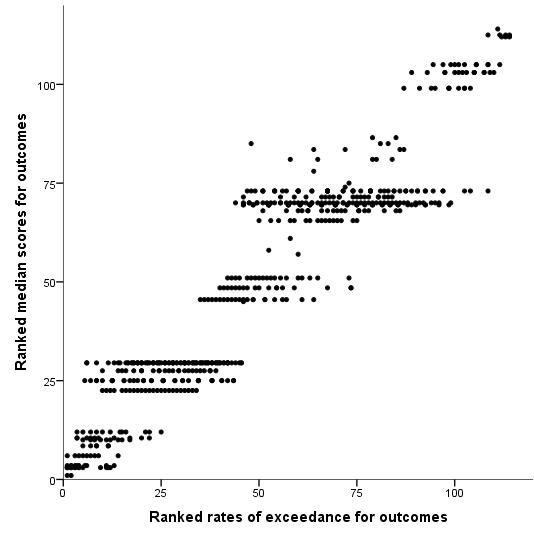
Figure S4** Comparison of ranked rates of exceedance and ranked median scores for outcomes across both Delphi projects

Median and rate of exceedance calculated for each outcome and then ranked within individual rounds of the two Delphi projects

**Table S3** Outcomes ranked highest by different summary statistics in final round of gastroschisis core outcomes set development

|  | Summary statistic | | |
| --- | --- | --- | --- |
|  | Mean | Median | Rate of Exceedance |
| 1 | Mortality | Mortality | Quality of life for the child |
| 2 | Quality of life for the child | Quality of life for the child | Mortality |
| 3 | Short bowel syndrome | Short bowel syndrome | Liver disease |
| 4 | Small bowel transplant | Small bowel transplant | Home parenteral nutrition |
| 5 | Liver disease | Liver transplantation | Short bowel syndrome |
| 6 | Abdominal compartment syndrome |  | Abdominal compartment syndrome |
| 7 | Bowel ischaemia |  | Bowel ischaemia |
| 8 | Home parenteral nutrition |  | Home total parenteral nutrition |

Top 8 outcomes listed to match size of final core outcomes set

Fewer than 8 outcomes listed by ranked median due to tie of median score outside top 8

**Table S4** Outcomes ranked highest by different summary statistics in final round of neonatal core outcomes set development

|  | Summary statistic | | |
| --- | --- | --- | --- |
|  | Mean | Median | Rate of Exceedance |
| 1 | Survival | Survival | Survival |
| 2 | Necrotising enterocolitis | Necrotising enterocolitis | Sepsis |
| 3 | Sepsis | Sepsis | Necrotising enterocolitis |
| 4 | Brain injury on imaging | Brain injury on imaging | Brain injury on imaging |
| 5 | Medical errors | Medical errors | Retinopathy of Prematurity |
| 6 | Retinopathy of Prematurity | Retinopathy of Prematurity | General gross motor ability |
| 7 | Visual impairment | Visual impairment | General cognitive ability |
| 8 | Quality of life | Quality of life | Ability to walk |
| 9 | Pain | Pain | Medical errors |
| 10 | Hearing impairment | Hearing impairment | Quality of life |
| 11 | Ability to walk | Ability to walk | Visual impairment |
| 12 | General cognitive ability | General cognitive ability | Hearing impairment |
|  |  | Suffering |  |
|  |  | Parental bonding with their baby |  |

Top 12 outcomes listed to match size of final core outcomes set

More than 12 outcomes listed by ranked median due to tie of median score in top 12
